# Bilharziosis in Girls and Women

**Published:** 1910-11-05

**Authors:** 


### Tropical Medicine.

### BILHARZIOSIS IN GIRLS AND WOMEN.

Three considerable fallacies in regard to the disease produced in residencs in certain parts of Africa, particularly Egypt, by the parasite bilharzia hæmatobia, have been disproved of recent years. These are, first, the view that the eggs passed per rectum have a lateral spine as distinct from the terminal spine of those passed in the urine; secondly, that males only are affected, or, at any rate, that hardly any females are attacked; and, thirdly, that the mode of infection is per urethram in persons addicted to swimming in fresh water.

The fact that girls and women may be affected by the disease in Egypt has been insisted upon by Dr. B. S. Elgood, Medical Officer in the Educational Department in Egypt. As the result of examination of the urine of girls in two middle-class schools in Egypt and of a certain number of female patients in Kasr-el-Ainy Hospital in Cairo he found as follows:—

The girls of the first school—forty in number, aged 12 to 16—were of the lower middle-class; their fathers were junior clerks, small shopkeepers, and teachers; the pupils were boarders at the school. All but one had lived in Cairo all their lives; the single exception came from the outskirts of a small provincial town, and she suffered from bilharzia. None of them had ever bathed in the Nile or in a canal; nor had any, except the girl from the provinces and one other, ever run about bare-legged in fields or on country roads. As far as they remembered they had all worn shoes and stockings when out in the streets of Cairo, and the Zoological Gardens were the only "country" place to which the other girls had ever been, and when there they had never been allowed to paddle or bathe.

All the homes of the above children were supplied by the Cairo Water Company from the Rod-el-Farag wells; and the water was drawn from taps and stored in earthenware zias to cool. Only one child stated that beside the water so stored some had also recently been drawn from a well in the courtyard of her father's house.

None of these children were so poor that they or their mothers washed clothes on the Nile bank, or fetched water thence, or washed vegetables there, as the women of the poorest classes all do.

The children did not complain of dysuria, nor would they admit that they ever passed blood either with urine or after it, though in many cases the urine was obviously smoky or blood-red. On examination of the urine of these forty girls, eleven, a percentage of 27.5, were found to be strongly infected by bilharzia.

As the source of infection at home was not quite clear, attention was turned to the water-supply at the school. The water here was the same as that supplied to the European quarter from the Rod-el-Farag well of the Cairo Water Company. It was delivered through metal tubing to a Berkefeld filter; the filtered water was then stored in a non-porous glazed earthenware receptacle, from which it was drawn off as required for use into small earthen jars. The water in the large receptacle and in small jars was examined for bilharzia embryos, and none were found. It would seem, therefore, that the drinking water was not a source of infection at the school. Nothing could be elicited as to any special ablutions which could have led to the infection. No other signs of bilharzia were present. Treatment by gram doses of extract of male fern noticeably diminished the number of eggs and the amount of blood in the urine.

In the second school the children belonged to a slightly higher class of life. Their ages varied from 6 to 13, and they came from various parts of Egypt. Though Egyptian girls are extremely prone to complain of any little ailment they become aware of, none of them complained of pain or bleeding on micturition. Of the thirty-nine boarders, eight, a percentage of 20.5, showed bilharzia eggs and blood in their urine. Of forty day girls, eleven showed bilharzia eggs in their urine, a percentage of 27.5. Though some of these children had lived in the country they were all of the well-to-do classes; they had none of them been allowed to paddle in canals, pools, or in the river.

It seems to be obvious from these statistics that quite a large number of Egyptian girls suffer from bilharzia. It is clear also that the view that was formerly accepted to the effect that the infection of males occurs per urethram is no longer tenable. It is possible that the drinking of water, or the eating of vegetables or fruit washed in dirty canals or rivers is a more likely source of the infection. It is a mere accident whether the spine of the ovum of the bilharzia parasite is at the end of the ovum or at its side; in the great majority of cases each ovum has a single spine at its smaller end; it is not distinctive of ova passed per rectum to have the lateral spine, for even those passed in the urine may occasionally exhibit it. Some hold that it is only when the adult female parasite is immature that the spines are lateral, the mature worm always passing ova with terminal spines.

